# The Root Extract of *Gentiana macrophylla* Pall. Alleviates Cardiac Apoptosis in Lupus Prone Mice

**DOI:** 10.1371/journal.pone.0127440

**Published:** 2015-05-18

**Authors:** Chih-Yang Huang, Tsai-Ching Hsu, Wei-Wen Kuo, Yi-Fan Liou, Shin-Da Lee, Da-Tong Ju, Chia-Hua Kuo, Bor-Show Tzang

**Affiliations:** 1 Graduate Institute of Basic Medical Science, China Medical University, Taichung, Taiwan; 2 Department of Health and Nutrition Biotechnology, Asia University, Taichung, Taiwan; 3 Institute of Microbiology and Immunology, Chung Shan Medical University, Taichung, Taiwan; 4 Clinical Laboratory, Chung Shan Medical University Hospital, Taichung, Taiwan; 5 Department of Biological Science and Technology, China Medical University, Taichung, Taiwan; 6 Department of Physical Therapy, Graduate Institute of Rehabilitation Science, China Medical University, Taichung, Taiwan; 7 Department of Neurological Surgery, Tri-Service General Hospital, National Defense Medical Center, Taipei, Taiwan; 8 Department of Sports Sciences, University of Taipei, Taipei, Taiwan; 9 Institute of Biochemistry and Biotechnology, Chung Shan Medical University, Taichung, Taiwan; 10 Department of Biochemistry, School of Medicine, Chung Shan Medical University, Taichung, Taiwan; Institute of Biochemistry and Biotechnology, TAIWAN

## Abstract

The roots of the perennial herb *Gentiana macrophylla* Pall. (GM) are known as Qinjiao, which has been used for centuries to treat systemic lupus erythematosus (SLE). However, little is known about the effects of GM on cholesterol-aggravated cardiac abnormalities in SLE, and the mechanisms thereof. This study investigates whether GM exhibits anti-apoptotic effects, focusing on the left ventricle (LV) of NZB/W F1 mice fed with high-cholesterol diet. The morphology and apoptotic status of ventricular tissues were determined by microscopy and Terminal deoxynucleotidyl transferase dUTP nick end labeling (TUNEL) assay. Levels of apoptotic biomarkers were determined by immunoblotting. The results thus obtained revealed that GM significantly reduced the cholesterol-aggravated apoptosis of LV in NZB/W F1 mice by suppressing both intrinsic and extrinsic apoptotic pathways. Additionally, GM significantly increased the cardiac insulin-like growth factors (IGF)-1 survival signaling and anti-apoptotic proteins in LV tissues. Accordingly, GM is considered to be beneficial in alleviating cholesterol-aggravated cardiac damage in SLE, and therefore constitute an alternative treatment for SLE patients with cardiac abnormalities.

## Introduction

Systemic lupus erythematosus (SLE) is known as an autoimmune disease with a variety of pathological manifestations, such as nephritis, arthritis, glomerulonephritis, pleuritis, and cardiovascular abnormalities [1]. Recent studies have reported that SLE patients have significant higher mortality and morbidity of cardiovascular diseases (CVD) [2–4]. Indeed, patients with SLE have remarkably high rates of coronary heart disorders [3, 5], mainly as pericarditis and myocarditis [6]. Besides, a 50-fold increased risk of myocardial infarction was reported in women with SLE aged between 44 and 50 [7], suggesting a significant incidence of cardiac disorder in some populations of SLE patients. Since apoptosis is strongly associated with various autoantibodies such as anti-phospholipid and anti-oxidized low-density lipoprotein antibodies in patients with SLE, engagement of these autoantibodies to self tissue is considered to activate the complement system, cell-mediated cytotoxicity and cardiomyocyte apoptosis [8–10]. Therefore, suppression of cardiac apoptosis is suggested to ameliorate autoantibody induced-cardiac injuries in SLE patients.

Gentianaceae comprises twenty-two species that are distributed mainly in China and includes two important medicinal plant species, *Gentiana* (GM) and *Swertia* (GS). *Gentiana macrophylla* Pall. (GM) is a perennial herb that belongs to the *Radix Gentianae macrophyllae* (Gentianaceae) [11] and is found only in the high alpine lands of the Tibetan Plateau at altitudes between 2400 and 3500 meters. The roots of GM are known as the traditional Chinese Medicine ‘‘Qinjiao”. For over 2000 years, GM has been prescribed for treating pain and inflammatory conditions [12]. GM contains some bitter compounds that have been extensively used in Chinese herbal medicine in the treatment of a wide range of diseases, including diabetes, apoplexy, paralysis and stomachache [13]. In an investigation of 58 patients with reflex sympathetic dystrophy (RSD), stagnation of vital energy and blood stasis, GM was demonstrated to have beneficial effects in the treatment of RSD by improving blood circulation and removing blood stasis [14]. GM has also been reported to alleviate both CCl_4_-induced and lipopolysaccharide (LPS)-induced liver injuries by suppressing the production of tumor necrosis factor (TNF) [15] and the anti-inflammatory activity that is observed in a carrageenan-induced paw edema model [12]. Notably, GM extract has also been used to treat rheumatoid arthritis and SLE [16–18], but its precise effects and underlying mechanisms remain unclear.

Studies of the use of alternative medicines to treat SLE are attracting increasing attention. According to a recent review of the literature, over 50% of patients with SLE have used complementary and alternative medicine (CAM) to alleviate symptoms and manage their health [19]. Although GM has been used for centuries to treat many inflammatory diseases, little is known about the effects of GM on lupus-associated cardiac abnormalities. This study investigates whether GM alleviates cholesterol-aggravated cardiac apoptosis in NZB/W F1 mice, with emphasis on the underlying mechanisms.

## Materials and Methods

### Preparation of *Gentiana macrophylla* Pall. extract and composition analysis

Extract of *Gentiana macrophylla* Pall. (GM) was prepared as described elsewhere [12]. The roots of GM were purchased from a domestic traditional herb store (Chen-Oriental-Lin Ginseng Medicine Store, Taishan District, New Taipei City, Taiwan), cut into small pieces and then ground into a powder. The GM powder was soaked in 80% ethanol at room temperature for one week. The solution was then centrifuged at 20000g for 30 min at 4°C to remove insoluble pellets. After it had been filtered through two layers of gauze (0.45 mm), the extract solution was lyophilized in a freeze-dry system and stored at −80°C until use. GM extract (198.9g, 23.8% yield w/w) was thereby obtained. Table 1 presents the obtained content of polyphenol in GM, revealing a final extract composition of 98.26 ± 3.99 (mg gallic acid equivalent (GAE)/g dry weight of GM extract) polyphenolic acid and 10.68 ± 1.01 (mg rutin equivalent (GAE)/g dry weight of GM extract) flavonoids.

**Table 1:** Major components of phenolic acids and flavonoids in GM extract.

Ingredients	Concentration (mg/g)	Percentage (%)
Gallic acid	4.36 ± 0.19	0.44 ± 0.09
Catechin	2.17 ± 0.21	0.22 ± 0.06
Gentisic acid	13.69 ± 0.76	1.37 ± 0.13
Caffeic acid	47.34 ± 1.98	4.73 ± 0.36
Epicatechin	ND	-
Ferulic acid	ND	-
Rutin	ND	-
Neohesperidin	ND	-
Quercetin	ND	-
Narigenin	ND	-
Luteolin	ND	-

All values are mean ± SD obtained by triplicate analyses.

GM: *Gentiana macrophylla Pall*.

ND: not determined.

### Mice and Diets

This study was approved by the Institutional Animal Care and Use Committee at Chung Shan Medical University (IACUC approval No. 1456). Animal welfare and experimental procedures were performed according to the NIH Guide for the Care and Use of Laboratory Animals. As described in our recent publications [20–22], female NZB/W F1 mice were purchased from Jackson Lab, USA and housed under supervision of the Institutional Animal Care and Use Committee at Chung Shan Medical University. The mice were kept in an animal room at 22°C with a 12/12 h light-dark cycle. Chow diet, soybean oil, and cholesterol were purchased (TestDiet Division, PMI Nutrition International/ Purina Mills LLC, Richmond, IN). The control diet comprised 93% rodent 5001 chow diet and 7% soybean oil. The cholesterol diet comprised 92% rodent 5001 chow diet, 7% soybean oil, and 1% cholesterol. The cholesterol/GM diet comprised 91% rodent 5001 chow diet, 7% soybean oil, 1% cholesterol, and 1% GM. Thirty female NZB/W F1 mice with an age of 12-weeks were divided into three groups (10 mice/group), which were separately given control, cholesterol, and cholesterol/GM diets for 12 weeks. Mice were sacrificed at the age of 24 weeks by CO_2_ asphyxiation. The heart tissues of the mice were then obtained and stored at -80°C until use.

### Hematoxylin-eosin staining

As described in detail previously [20–22], heart samples of the animals were excised and soaked in formalin and covered with wax. The waxed tissue blocks were cut into 5 μm-thick sections and slices were prepared by deparaffinization and dehydration. The sections were passed through a series of graded alcohols (100%, 95% and 75%) 15 min in each. The slices were then dyed with hematoxylin. Following gentle rinsing in water, each slice was soaked for 15 min in each of 85% alcohol, 100% alcohol I and II in that order. Finally, each slice was soaked with Xylene I and Xylene II. Photomicrographs were obtained using Zeiss Axiophot microscopes.

### TUNEL Assay

Apoptotic cells were detected by TUNEL assay as described in detail previously [20, 23]. The left ventricular tissues from NZB/W F1 mice were embedded into Optimal Cutting Temperature (OCT) compound (Tissue-Tek, Miles Inc., Elkhart, IN) and snap-frozen in liquid nitrogen. The frozen tissue blocks were cut into 5 μm-thick sections and fixed in 4% paraformaldehyde (Sigma-Aldrich Co., St. Louis, MO, USA) in 0.1 M phosphate-buffered saline (PBS), pH 7.4, for 20 min at room temperature. After they were washed for 30min with 0.1M PBS, the tissue sections were incubated with 3% H_2_O_2_ in methanol for 10 min at room temperature. The TUNEL reaction mixture was freshly prepared following the manufacturer’s instructions (RocheApplied Science, Inc., United States), and a total volume of 100 μL of terminal deoxytransferase reaction mixture was incubated with the tissue sections for 1 h at room temperature in the dark. The tissue sections were then rinsed with 0.1M PBS that contained DAPI and observed under a fluorescence microscope. The number and percentage of TUNEL-positive cells were determined by counting 1x10^3^ cardiac cells in five randomly selected fields. All measurements were made blindly using at least three independent animals.

### Preparation of tissue extract and determination of protein

All procedures were performed at 4°C in a cold room as described in detail previously [21–22]. The left ventricle tissues that were obtained from NZB/W F1 mice were homogenized in 600 μl PRO-PREP solution (iNtRON Biotech, Korea) by 30 strokes using a Dounce Homogenizer (Knotes Glass, Vineland, NJ). The homogenates were centrifuged at 13,000 rpm for 10 min at 4°C and the supernatants were then stored at −80°C until use. The concentration of protein in the tissue extracts was determined using method that has been described elsewhere [24].

### Western blotting

Protein samples were separated in 10% or 12.5% SDS-PAGE and electrophoretically transferred to a nitrocellulose membrane (Amersham Biosciences, Piscataway, NJ, USA) as described in detail previously [20–22, 25]. After blocking with 5% non-fat dry milk in PBS, antibodies against TNF-α receptor, TNF-α, Fas, Fas-Associated protein with Death Domain (FADD), Bax, caspase-8, caspase-9, caspase-3, IGF-1R, p-PI3K, p-AKT(Ser473), Bcl2, Bcl-xL (Santa Cruz Biotechnology, CA, USA) and α-Tubulin (Upstates, Charlottesville, VA, USA) were diluted in PBS with 2.5% BSA and incubated for 1.5 h with gentle agitation at room temperature. The membranes were washed twice with PBS-Tween for 1 h and secondary antibody that was conjugated with horseradish peroxidase (HRP) (Santa Cruz Biotechnology, Santa Cruz, CA, USA) was added. Pierce's Supersignal West Dura HRP Detection Kit (Pierce Biotechnology Inc., Rockford, IL) was used to detect antigen–antibody complexes, which were quantified by densitometry (Appraise, Beckman-Coulter, Brea, CA, USA).

### Statistical Analysis

All statistical analyses were performed using SPSS 10.0 software (SPSS Inc., Chicago, IL) as described in our recent publications [20–22]. Experiments were performed in triplicate. Statistical analyses involved analysis of variance plus posterior multiple comparison test to test difference. P<0.05 indicates statistical significance and is shown as a symbol.

## Results

### Cardiac histopathological changes in NZB/W F1 mice fed with different dietary supplements

**Fig 1:**
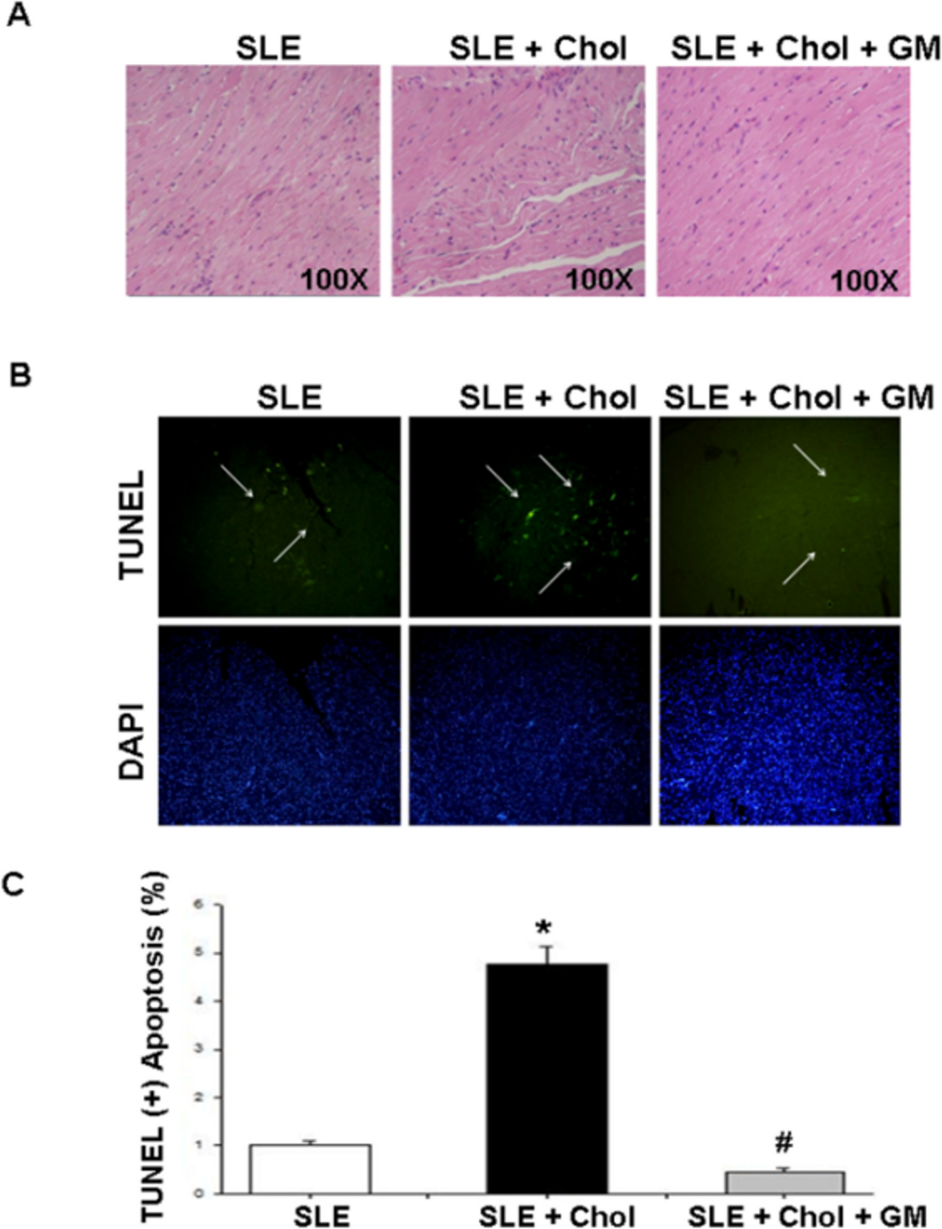
Cardiac histopathological changes in NZB/W F1 mice fed with different dietary supplements. (A) Histopathological analysis of cardiac tissue sections with hematoxylin and eosin staining and (B) representative stained apoptotic cells of cardiac sections from NZB/W F1 mice fed with different supplementations, as obtained by TUNEL assay. (C) Percentages of apoptotic cells were calculated. Images of myocardial architecture were magnified 100 times. Bars represent number of TUNEL-positive cells as a percentage of total number of cells (10 scope field count for each mice in each group) and values are expressed as mean ±SD. * and # indicate significant differences relative to SLE and SLE + cholesterol groups, respectively. Similar results were obtained in triplicate experiments. SLE: systemic lupus erythematosus; Chol: cholesterol; GM: *Gentiana macrophylla* Pall.

To investigate the variation of hearts in NZB/W F1 mice that were fed with different dietary supplements, physiological changes of left ventricles were measured and histopathological analysis of the left ventricles were performed by hematoxylin and eosin (HE) staining and TUNEL assay. The ratios of the left ventricular weight (LVW) to the whole heart weight (WHW) and of the LVW to the tibia length were significantly lower for NZB/W F 1 mice that were fed with the cholesterol diet than for those of the control group. Conversely, the NZB/W F1 mice that had been fed with the cholesterol/GM diet had significantly higher LVW to WHW and LVW to tibia length ratios than did the cholesterol group (Table 2). The ventricular myocardium in the cholesterol group exhibited a more abnormal architecture than the control group, revealing cardiomyocyte disarray and a larger interstitial space. Conversely, less abnormal architecture was observed in the cholesterol/GM group was observed than in the cholesterol group (Fig 1A). Additionally, a significantly increased number of TUNEL-positive cardiac cells were detected in the cholesterol group, and significantly fewer TUNEL-positive cardiac cells were observed in the cholesterol/GM group (Fig 1B). The mean percentages of TUNEL-positive cardiac cells in the control, cholesterol, and cholesterol/GM groups were 1.18±0.17, 4.99±0.68, 0.38±0.09, respectively (Fig 1C).

**Table 2:** Physiological change of left ventricles.

Items	SLE	SLE + Cholesterol	SLE + Cholesterol + GM
Body weight (BW), g	41.43±1.13	38.28±0.11	41.48±3.39
Whole heart weight (WHW), g	0.155±0.009	0.159±0.015	0.155±0.011
Left ventricular weight (LVW), g	0.111±0.006	0.091.±0.052	0.112±0.009
WHW/BW (×10^4^)	37.43±0.0003	41.6.±0.0004	37.6±0.0005
LVW/BW (×10^4^)	26.78±0.0002	23.76±0.0014	27.11±0.0004
LVW/WHW, g	0.725±0.024	0.554±0.293*	0.721±0.040^#^
WHW/Tibia, g/mm (×10^4^)	79.7±0.0005	80.82±0.0007	79.55±0.0005
LVW/Tibia, g/mm (×10^4^)	57.0±0.0004	45.99±0.0026*	57.33±0.0004^#^

Values are mean ± standard deviation.

* Indicates P < 0.05,

# compared with SLE and SLE + Cholesterol

### Changes of Fas-related and mitochondrial-dependent components in the left ventricles of NZB/W F1 mice fed with different dietary supplements

**Fig 2:**
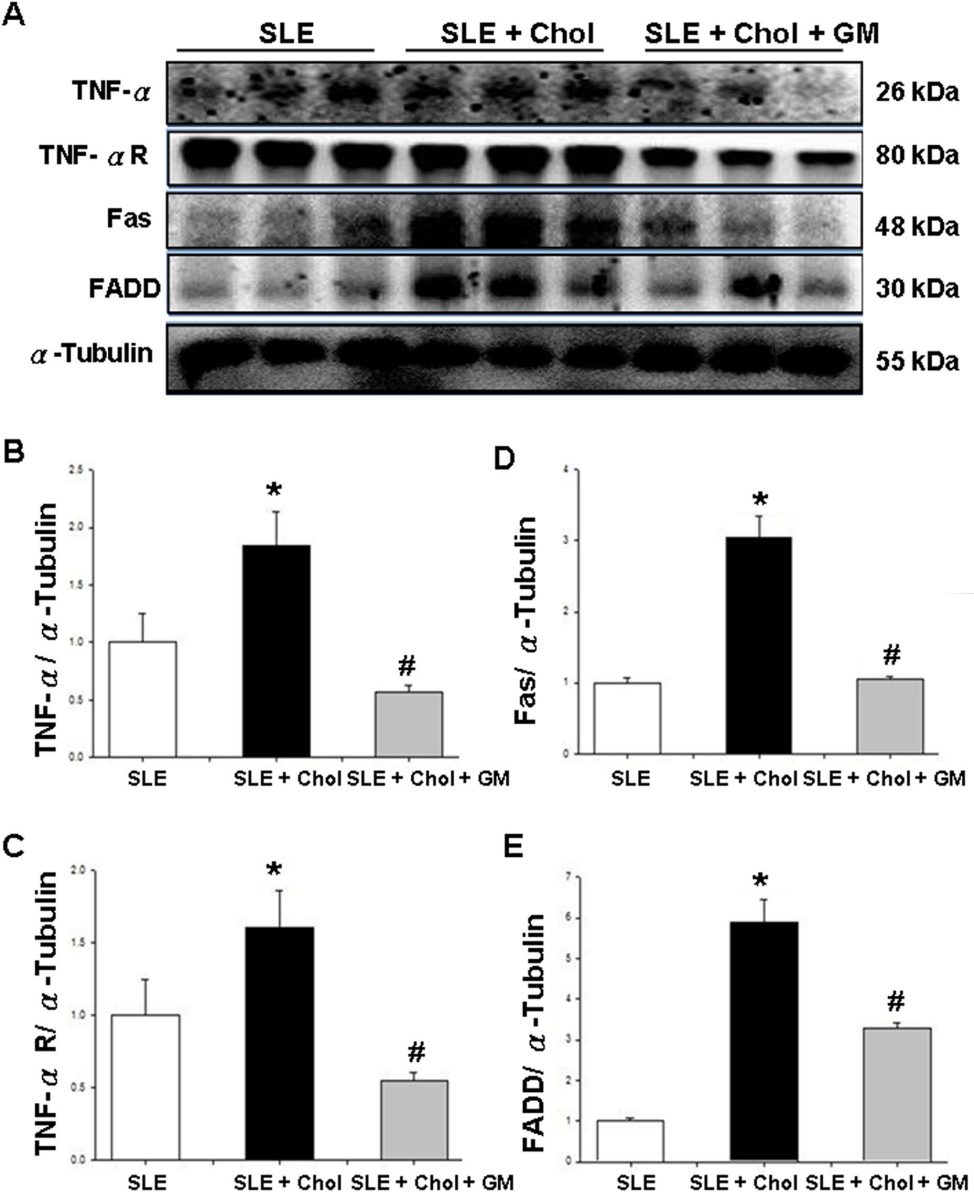
Variation of Fas-related components in the left ventricular tissues of NZB/W F1 mice fed with different dietary supplements. (A) Expressions of TNF-α, TNF-α receptor, Fas and FADD in left ventricles of hearts from NZB/W F1 mice that were fed with different supplementations, measured by Western blotting analysis. α-Tubulin served as an internal control. Bars represent quantities of (B) TNF-α, (C) TNF-α receptor, (D) Fas and (E) FADD relative to amount of α-Tubulin, respectively. * and # indicate significant differences relative to SLE and SLE + cholesterol groups, respectively. Similar results were obtained in triplicate experiments. SLE: systemic lupus erythematosus; Chol: cholesterol; GM: *Gentiana macrophylla* Pall.

**Fig 3:**
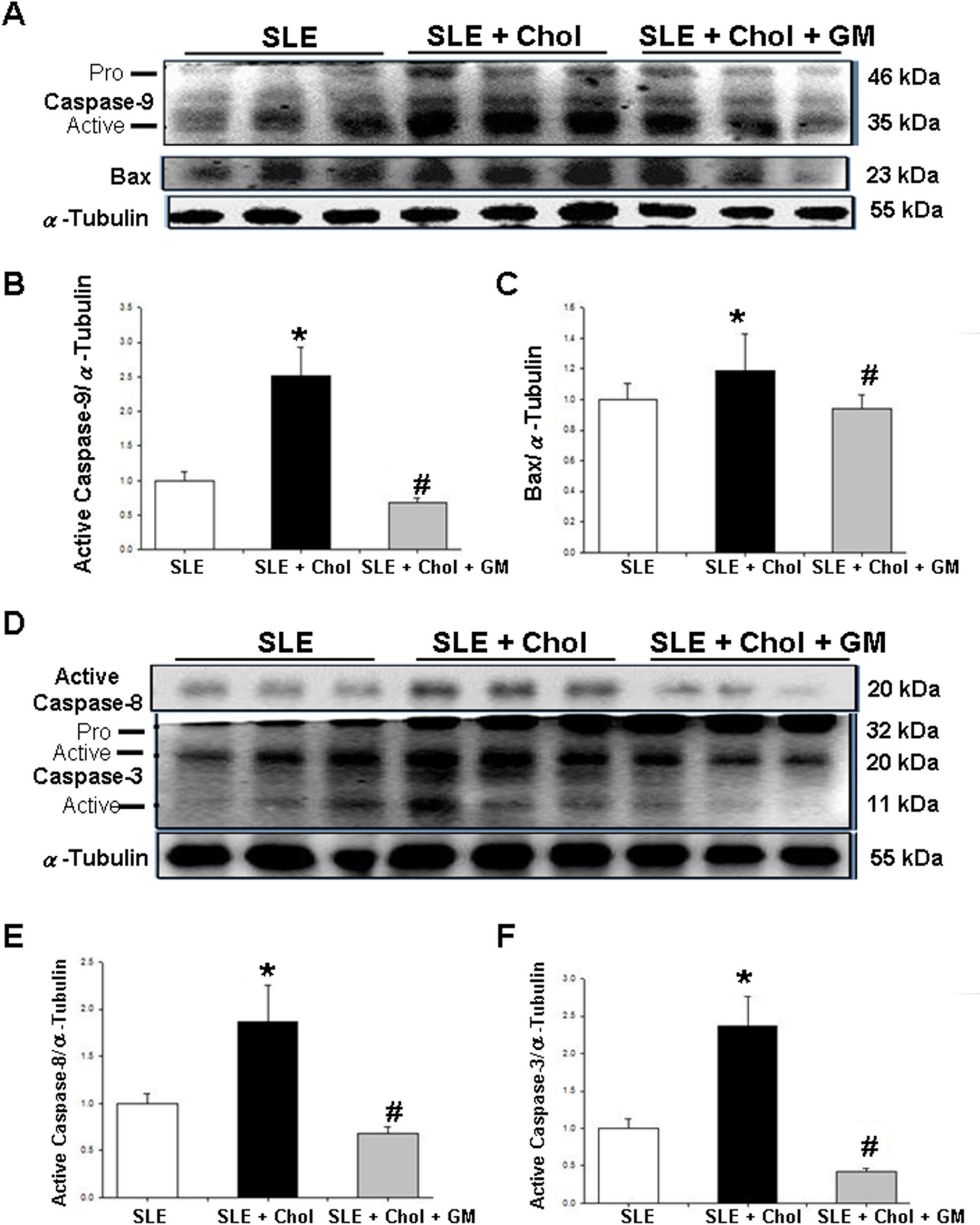
Variations in mitochondrial-dependent components in left ventricular tissues of NZB/W F1 mice that were fed with different dietary supplements. (A) Expressions of active caspase-9 and Bax in left ventricles of hearts of NZB/W F1 mice fed with different supplements, as measured by Western blotting analysis. α-Tubulin was used as an internal control. Bars represent quantities of (B) active caspase-9 (35kDa) and (C) Bax relative to that of α-Tubulin. (D) Expressions of active caspase-8 and caspase-3 in left ventricles of hearts from NZB/W F1 mice fed with different supplements, as measured by Western blotting analysis. α-Tubulin was used an internal control. Bars represent quantities of (E) active caspase-8 and (F) active caspase-3 (20kDa) proteins relative to quantity of α-Tubulin, respectively. * and # indicate significant differences relative to SLE or SLE + cholesterol groups, respectively. Similar results were obtained in triplicate experiments. SLE: systemic lupus erythematosus; Chol: cholesterol; GM: *Gentiana macrophylla* Pall.

To study the effects of different dietary supplements on cardiac apoptosis in NZB/W F1 mice, Fas-related and mitochondrial-dependent components were detected by Western blotting. The amounts of Fas death receptor-related components, including Fas, FADD, TNF-α and the TNF-α receptor, was significantly higher in the left ventricles of the NZB/W F1 mice that were fed with cholesterol dietary supplement than in those of the control group (Fig 2A). Conversely, the amount of these Fas death receptor-related components was significantly lower in the left ventricles of NZB/W F1 mice that were fed with cholesterol/GM dietary supplement than in those of the cholesterol group (Fig 2A). The ratios of Fas, FADD, TNF-α and TNF-α receptors to α-Tubulin were calculated and shown in Fig 2B–2E, respectively. Additionally, expressions of mitochondrial-dependent apoptotic components, such as caspase-9 and Bax, were detected. Significantly increased amounts of activated caspase-8 and Bax were detected in the left ventricles of NZB/W F1 mice that were fed with the cholesterol dietary supplement than in those of the control group, whereas significantly less activated caspase-9 and Bax were observed in the cholesterol/GM group than in the cholesterol group (Fig 3A). Fig 3B and 3C present the amounts of activated caspase-9 and Bax proteins relative to the quantity of α-Tubulin, respectively (Fig 3B and 3C). Accordingly, the amounts of activated caspase-8 and caspase-3, the downstream apoptotic molecules, were significantly higher in the cholesterol group than in the control group. Conversely, activated caspase-8 and caspase-3 levels were significantly lower in the cholesterol/GM group than in the cholesterol group (Fig 3D). Fig 3E and 3F present the amounts of activated caspase-8 and caspase-3 proteins, respectively, relative to that of α-Tubulin.

### Expression of cardiac survival signaling in the left ventricles of NZB/W F1 mice fed with different dietary supplements

**Fig 4:**
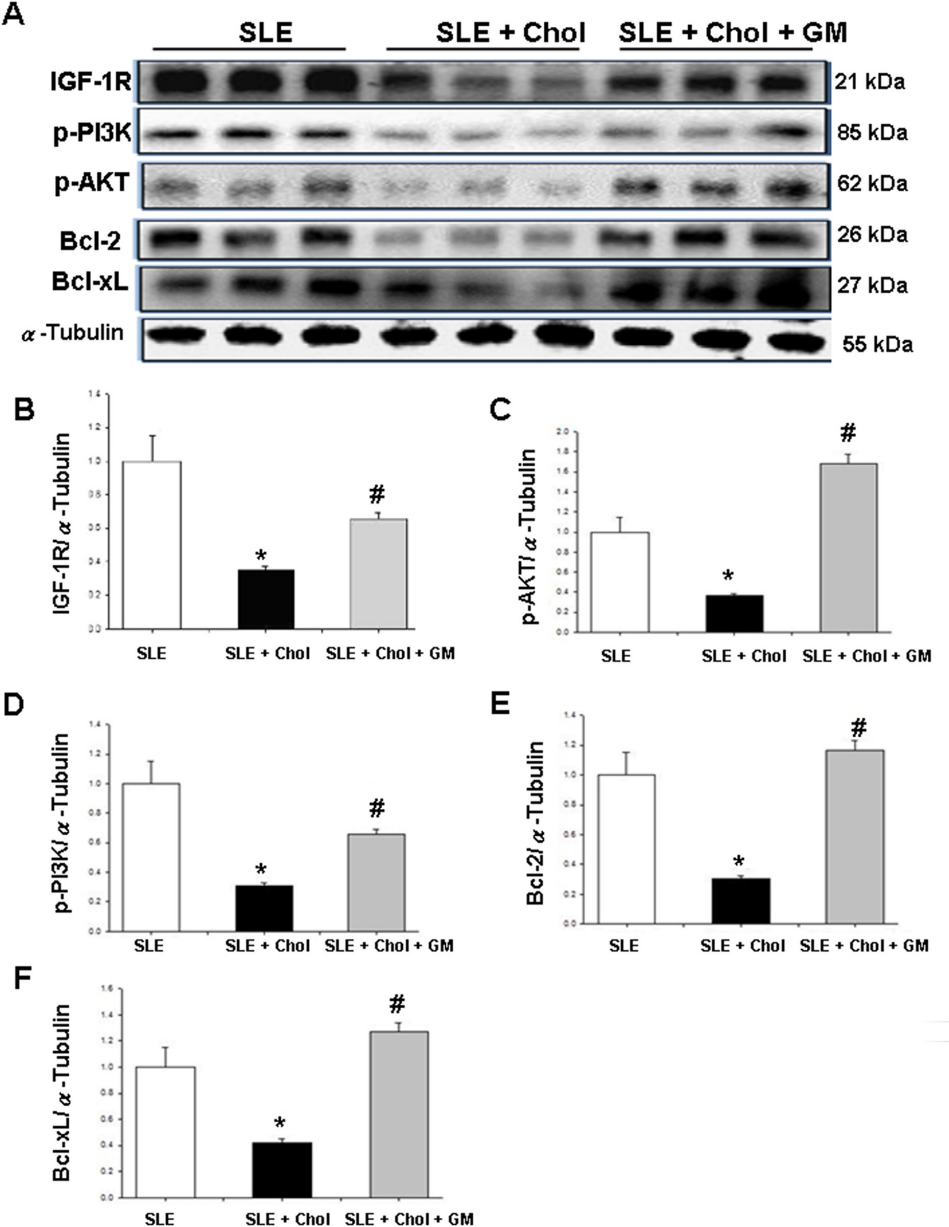
Expression of cardiac survival signal in left ventricular tissues of NZB/W F1 mice fed with different dietary supplements. (A) Expressions of IGF-1R, p-PI3K, p-AKT(Ser473), Bcl-2 and Bcl-xL in left ventricles of hearts from NZB/W F1 mice fed with different supplements, as measured by Western blotting analysis. α-Tubulin used as an internal control. Bars represent quantities of (B) IGF-1R, (C) p-PI3K, (D) p-AKT(Ser473), (E) Bcl-2 and (F) Bcl-xL proteins relative to quantity of α-Tubulin. * and # indicate significant differences relative to SLE and SLE + cholesterol groups, respectively. Similar results were obtained in triplicate experiments. SLE: systemic lupus erythematosus; Chol: cholesterol; GM: *Gentiana macrophylla* Pall.

To study further the variation of cardiac survival signaling components in NZB/W F1 mice, the levels of IGF-1R, p-PI3K and p-AKT(Ser473) proteins were investigated. The expressions of IGF-1R, p-PI3K and p-AKT(Ser473) were significantly lower in the left ventricles of NZB/W F1 mice that had been fed with cholesterol dietary supplements than in the control group (Fig 4A). Conversely, IGF-1R, p-PI3K and p-AKT(Ser473) protein levels were significantly higher in the left ventricles of the NZB/W F1 mice that had been fed with cholesterol/GM dietary supplements than in the cholesterol group (Fig 4A). Fig 4B–4D present the quantities of IGF-1R, p-PI3K and p-AKT(Ser473) relative to the amount of α-Tubulin. Meanwhile, levels of Bcl-2 and Bcl-xL, the anti-apoptotic proteins, were significantly lower in the left ventricles of the NZB/W F1 mice that were fed with cholesterol dietary supplements. Conversely, levels of Bcl-2 and Bcl-xL were significantly higher in the left ventricles of the NZB/W F1 mice that had been fed with cholesterol/GM dietary supplements than in the cholesterol group (Fig 4A). Fig 4E and 4F present the amounts of Bcl-2 and Bcl-xL proteins, respectively, relative to that of α-Tubulin.

## Discussion

An increasing number of studies are reporting that SLE patients use complementary and alternative medicines (CAM) to alleviate symptoms and improve their quality of life (QOL) [19]. Although GM extract, a popular traditional Chinese medicine, has been used to treat rheumatoid arthritis and SLE [17–18], the precise effects and their underlying mechanisms remain unclear. In this work, GM extract is demonstrated to reduce cholesterol-aggravated apoptosis in the left ventricular tissues of NZB/W F1 mice and to induce cardiac survival signaling. This finding provides a clue to the possible mechanism of GM and suggests the therapeutic potential of GM against cardiac apoptosis associated with SLE.

Extrinsic and intrinsic pathways are both important in triggering apoptosis [26]. The extrinsic pathway begins outside the cell with the activation of pro-apoptotic receptors on the cell surface. Engagements of pro-apoptotic receptors and their ligands, such as TNF-α/TNF-α receptors and Fas/Fas receptors, cause those receptors to cluster and form a death-inducing signaling complex (DISC). After the activation of DISC, caspases, such as caspase-8 and caspase-3, are initiated to induce DNA fragmentation [27]. The intrinsic pathway, which is the so-called mitochondrial-dependent pathway, is commonly initiated in response to intracellular signals such as DNA damage, loss of cell-survival factors, or excessive oxidative stress. Typically, pro-apoptotic proteins such as cytochrome c and Bax are released from the mitochondria and activate caspase proteases, such as caspase-9, and trigger apoptosis [28]. The intrinsic pathway is known to depend on the balance of activity between pro- and anti-apoptotic signals of the Bcl-2 family [29]. Relevant research shows that in this cascade, anti-apoptotic proteins such as Bcl-2 and Bcl-xL antagonize Bax and Bak by binding to their BH3 domains [30]. Our previous study demonstrated that a high-cholesterol diet reduced the cardiac IGF-1 survival components and increased extrinsic and intrinsic apoptotic signaling in the LV tissues of NZB/W-F1 mice [20]. The experimental results in this study demonstrate that GM not only alleviates the extrinsic and intrinsic apoptotic signaling but also increases cardiac IGF-1 survival signaling and the amounts of anti-apoptotic components such as Bcl-2 and Bcl-xL. These findings indicate that the inhibition of both intrinsic and extrinsic pathways of apoptosis is a response to exposure of LV tissues to GM as a result of elevated IGF-1 signaling and anti-apoptotic proteins. However, this study has some limitations, which must be addressed. Previous investigations have reported the improvements by GM of nephropathy, arthralgia and erythema in SLE patients [16–17]. However, the effects of GM on cardiac function in SLE patients have not yet been investigated. Although this work is the first to demonstrate the advantageous effects of GM on cardiac apoptosis in LV tissues of lupus-prone mice, its findings do not directly reveal the effect of GM on cardiac function in patients with SLE. Therefore, further investigation, including transthoracic echocardiograms, is required to confirm the effects of GM on cardiac function in patients with SLE.

An increasing number of studies are reporting that over half of patients with SLE use complementary and alternative medicine (CAM) treatments to alleviate symptoms and manage their health [19]. Many groups have chemically investigated roots of *Gentiana macrophylla* Pall., a traditional Chinese drug with a long history of use in treating jaundice, hepatitis, constipation, pains and rheumatism [16,31–33]. Although most of the constituents of GM have been identified [34–35], information about the functional constituents and their mechanisms is limited, especially in relation to autoimmune diseases. In an SD-rat model that was treated with LPS, gentianine, a major component of GM, exhibits anti-inflammatory activity through the prevention of the immune cells, including macrophages, from producing TNF-a and IL-6, pro-inflammatory cytokines [36]. This effect is anti-inflammatory action considerably potent in cases of rheumatoid arthritis [18]. As presented in this work, GM extract attenuates lupus-associated cardiac apoptosis in lupus-prone mice by down-regulating TNF-α/TNF-α receptors and Fas/FADD and reducing amounts of activated caspase-9, Bax, activated caspase-8 and activated caspase-3. Meanwhile, increased amounts of cardiac IGF-1R survival components following the activation of p-PI3K and p-AKT(Ser473) and elevated expressions of anti-apoptotic proteins, Bcl-2 and Bcl-xL, were detected upon the administration of GM extract. These findings demonstrate that the anti-apoptotic effects of GM extract in the left ventricles of NZB/W F1 mice is attributable to the gentisic acid or other polyphenol compounds with anti-inflammation property. However, further investigations are needed to identify the functional constituents of GM extracts.

## Conclusion

Altogether, this study provides evidences that the treatment of GM extract significantly alleviates cholesterol-aggravated cardiac apoptosis in left ventricle tissues in NZB/W F1 mice perhaps by augmenting the cardiac IGF-1 survival signal through the phosphorylation of PI3K and AKT and the inhibition of both extrinsic and intrinsic apoptosis signals. These findings indicate that GM extract may protect LV tissues from cholesterol-aggravated apoptosis in lupus-prone mice and it offers a potential therapy against CVD in SLE patients.
